# Nontraditional Roles of Magnesium Ions in Modulating Sav2152: Insight from a Haloacid Dehalogenase-like Superfamily Phosphatase from *Staphylococcus aureus*

**DOI:** 10.3390/ijms25095021

**Published:** 2024-05-04

**Authors:** Jaeseok Bang, Jaehui Park, Sung-Hee Lee, Jinhwa Jang, Junwoo Hwang, Otabek Kamarov, Hae-Joon Park, Soo-Jae Lee, Min-Duk Seo, Hyung-Sik Won, Seung-Hyeon Seok, Ji-Hun Kim

**Affiliations:** 1 College of Pharmacy, Chungbuk National University, Cheongju 28160, Republic of Korea; mycrong11@chungbuk.ac.kr (J.B.); sweetpea0609@g.cbnu.ac.kr (J.P.); suzukaze@naver.com (S.-H.L.); zmdidi@naver.com (J.J.); hjw0788@chungbuk.ac.kr (J.H.); kamarovotabek@gmail.com (O.K.); gowns0419@chungbuk.ac.kr (H.-J.P.); sjlee@chungbuk.ac.kr (S.-J.L.); 2 Department of Molecular Science and Technology, Ajou University, Suwon 16499, Republic of Korea; mdseo@ajou.ac.kr; 3 College of Pharmacy, Research Institute of Pharmaceutical Science and Technology (RIPST), Ajou University, Suwon 16499, Republic of Korea; 4 Department of Biotechnology, Research Institute (RIBHS), College of Biomedical and Health Science, Konkuk University, Chungju 27478, Republic of Korea; wonhs@kku.ac.kr; 5 BK21 Project Team, Department of Applied Life Science, Graduate School, Konkuk University, Chungju 27478, Republic of Korea; 6 College of Pharmacy, Interdisciplinary Graduate Program in Advanced Convergence Technology and Science, Jeju National University, Jeju 632433, Republic of Korea

**Keywords:** *Staphylococcus aureus*, crystal structure, haloacid dehalogenase-like hydrolase, phosphatase, melting temperature, function of magnesium ion, functional replacement

## Abstract

Methicillin-resistant *Staphylococcus aureus* (MRSA) infection has rapidly spread through various routes. A genomic analysis of clinical MRSA samples revealed an unknown protein, Sav2152, predicted to be a haloacid dehalogenase (HAD)-like hydrolase, making it a potential candidate for a novel drug target. In this study, we determined the crystal structure of Sav2152, which consists of a C2-type cap domain and a core domain. The core domain contains four motifs involved in phosphatase activity that depend on the presence of Mg^2+^ ions. Specifically, residues D10, D12, and D233, which closely correspond to key residues in structurally homolog proteins, are responsible for binding to the metal ion and are known to play critical roles in phosphatase activity. Our findings indicate that the Mg^2+^ ion known to stabilize local regions surrounding it, however, paradoxically, destabilizes the local region. Through mutant screening, we identified D10 and D12 as crucial residues for metal binding and maintaining structural stability via various uncharacterized intra-protein interactions, respectively. Substituting D10 with Ala effectively prevents the interaction with Mg^2+^ ions. The mutation of D12 disrupts important structural associations mediated by D12, leading to a decrease in the stability of Sav2152 and an enhancement in binding affinity to Mg^2+^ ions. Additionally, our study revealed that D237 can replace D12 and retain phosphatase activity. In summary, our work uncovers the novel role of metal ions in HAD-like phosphatase activity.

## 1. Introduction

After the initial isolation of *Staphylococcus aureus* (*S. aureus*) by Rosenbach, it became recognized as a significant human pathogen and adapted to the healthcare environment [1]. *S. aureus* is associated with various diseases including endocarditis, bacteremia, osteomyelitis, sepsis, and skin and soft tissue infections [2]. Resistance to treatment emerged shortly after the use of penicillin for *S. aureus* in the 1940s. Methicillin-resistant *S. aureus* (MRSA) isolates were first discovered in the 1960s, just a year after the introduction of methicillin. Methicillin resistance is conferred by *mecA*, which encodes the penicillin-binding protein 2A responsible for crosslinking peptidoglycans in the bacterial cell wall and is located on the staphylococcal cassette chromosome mec [3,4].

While the pandemic strain of *S. aureus* has not been identified, MRSA infections have a global presence. Initially, healthcare-associated MRSA was frequently detected, but in recent years, community-associated MRSA has rapidly spread in the community [5,6,7,8]. *S. aureus* is typically classified based on its genetic characteristics, which can provide insights for the development of new antibiotic agents against MRSA [9]. Multilocus sequence typing (MLST), a genetic analysis method, is useful for characterizing bacterial isolates using the sequences of internal fragments of seven housekeeping genes [10,11]. Genomic analysis has revealed significant differences among various *S. aureus* strains, highlighting the need to identify common virulence factors and antigens for antibiotic development [12].

Yamamoto et al. conducted an analysis of secretomes from two clinical isolates of European community-associated MRSA and identified a total of 174 distinct proteins present in both isolates [13]. In terms of novel antibiotic development, previously uncharacterized proteins can serve as candidates for unique drug targets through structure–function analysis [14]. In this context, an unknown protein called Sav2152, predicted to be a haloacid dehalogenase (HAD)-like hydrolase, among the identified proteins in the secretome, is expected to be a novel candidate for drug targeting.

HAD-like hydrolases belong to a superfamily of enzymes that remain largely uncharacterized. Many characterized members of this superfamily possess phosphatase, phosphoglucomutase, phosphonatase, and dehalogenase activities [15]. Individual bacterial genomes may contain 10 to 20 HAD genes. Although HAD-like hydrolases exhibit relatively low overall sequence identity (15–30% identity), they possess four conserved short sequence motifs. Most characterized HAD-like hydrolases demonstrate phosphatase activity. Phosphoryl-transfer reactions are involved in various biological processes across all organisms and often play pivotal roles in bacterial survival. Additionally, obtaining structural information at the atomic resolution enables efficient target validation and facilitates the development of novel agents through structure-based drug design.

Therefore, our study aimed to investigate the structural properties and biological function of Sav2152 from the *S. aureus* Mu50 strain. While several structures of HAD-like phosphatases have been reported, the detailed mechanism of Sav2152 was expected to differ owing to its low sequence similarity to others. A comparative analysis of HAD-like hydrolase structures can provide valuable insights into the biological processes of these proteins. Here, we present the successful determination of the monomeric crystal structure of Sav2152 at 1.9 Å resolution. Moreover, as Sav2152 exhibits metal-dependent phosphatase activity, the role of key residues in the protein will be discussed based on the structural information.

## 2. Results

### 2.1. Oligomeric State of Sav2152

**Figure 1:**
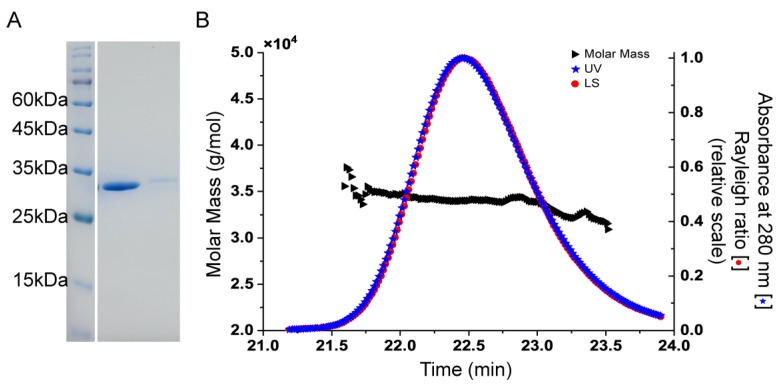
Oligomeric state of Sav2152. (**A**) Representative SDS-PAGE analysis showing single protein band of soluble protein and crystals in line 2 and 3, respectively. (**B**) Representative SEC-MALS analysis indicates that experimental molecular mass of purified Sav2152 is 34.4 kDa, corresponding to monomeric form.

Recombinant Sav2152 was successfully overexpressed using E. coli and purified (Figure 1A). HAD phosphatases have been reported to exist as monomers or polymers, such as PH1421 from Pyrococcus horikoshii OT3 and TA0175 from Thermoplasma acidophilum [16,17]. Therefore, the oligomeric state of Sav2152 was examined using SEC-MALS analysis. The representative Sav2152 showed a single elution peak with a molecular weight of 33.9 kDa, which is close to its theoretical monomeric molecular weight of 34.4 kDa (Figure 1B). This result clearly supports the conclusion that Sav2152 exists in a monomeric state.

### 2.2. Overall Crystal Structure of Sav2152

**Figure 2:**
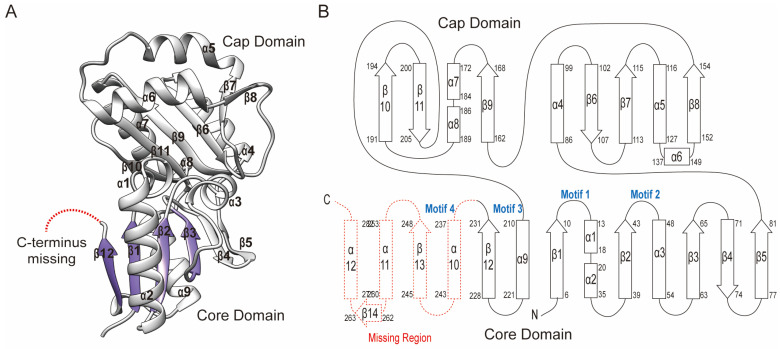
The crystal structure of Sav2152. (**A**) The ribbon representation of Sav2152. The structure is divided into two domains: the core domain and cap domain. The Rossmann fold is distinguished by color. α-helices and β-strands are individually labeled. There is a missing part in the C-terminus. This figure was produced using Chimera [18]. (**B**) A topology diagram of the secondary structure. Squares and broad arrows denote α-helices and β-strands, respectively. The four signature motifs are located in the space between the core domain and the cap domain.

To understand the structure–function relationship, the crystal structure of Sav2152 (PDB ID: 7XHZ) was determined using the molecular replacement (MR) method with the modeling structure generated by the Alphafold2 program. The structure of Sav2152 (res. 3-233) was refined at a resolution of 1.9 Å against the native data set. Data collection and refinement statistics are shown in Table 1. The crystal structure of Sav2152 was arranged sequentially in the order β1-α1-α2-β2-α3-β3-β4-β5-α4-β6-β7-α5-α6-β8-β9-α7-α8-β10-β11-α9-β12, lacking the C-terminus region (res. 234-285) (Figure 2). However, the absence of the C-terminus in the structure was not due to protein truncation, as demonstrated by the SDS-PAGE gel analysis of the protein crystal (Figure 1A). The absence of the C-terminus is probably due to a high degree of disorder of this region in the crystals. The HAD phosphatase superfamily generally shares the modified Rossmann fold, which forms a three-stacked α/β sandwich comprising repeating βαβ units. The central sheet of the general HAD phosphatase consists of at least five parallel strands arranged sequentially in a '54123" order. Unfortunately, the crystal structure of Sav2152 lacks a partial structure of the Rossmann fold. According to the model structure predicted from Alphafold2, β12 would be followed by α10-β13-α11-β14-α12, where β13 belongs to the Rossmann fold (Figure S1). The structure of Sav2152 is composed of two subdomains: a core domain (res. 1-81, 210-231) and a cap domain (res. 86-205). Based on the location of the cap domain between motif II and motif III, Sav2152 belongs to the C2 cap family.

### 2.3. Structural Comparison with Homology

**Figure 3:**
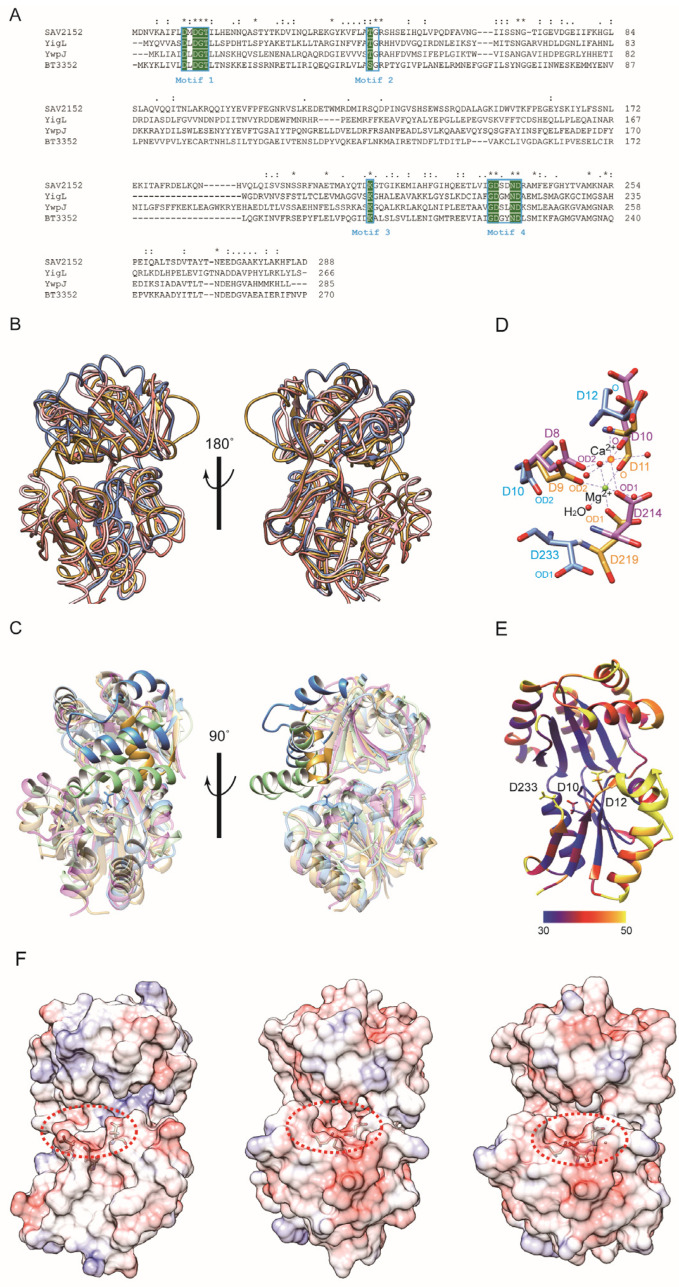
A structural analysis of Sav2152. (**A**) The multiple sequence alignment of Sav2152 and sequential homologs. The four signature motifs are enclosed in boxes. The conserved active site residues for phosphatase activities are highlighted in green. An asterisk (*), a colon (:), and a period (.) above the alignment indicate fully conserved residue, groups of strongly similar properties, and groups of weakly similar properties, respectively. The protein sequences were aligned and edited using ClustalW [21]. (**B**) A structural comparison of Sav2152 (blue) with YigL (PDB ID: 3pgv) (pink), BT3352 (4dwo) (gold), and YwpJ (1nrw) (green). (**C**) Diverse structures in the cap domain of Sav2152, YigL, BT3352, and YwpJ, colored in blue, pink, gold, and green, respectively. (**D**) A comparison of the key residues for metal binding. Sav2152, BT3352, and YigL are colored blue, gold, and pink, respectively. Three aspartic acids of BT3352 and YigL coordinate with Mg^2+^ and Ca^2+^, respectively. (**E**) Thermal parameter (B-factor) distribution in Sav2152. The B-factors are depicted on the structure from dark blue (lowest B-factor) to yellow (highest B-factor). Three key residues for metal binding are presented on the structure. (**F**) An electro-potential map of Sav2152 (**left**), BT3352 (**middle**), and YwpJ (**right**). The putative active sites are indicated by a dotted circle.

Since the protein structure lacks a substrate, metal ion, and C-terminus, further investigations were performed by comparing the structure with other structural homologs using the DALI search program [19]. The DALI result showed that the structure of YigL from Klebsiella pneumonia (PDB ID: 3pgv) highly resembles that of Sav2152, with a z score of 17.3, followed by YwpJ (1nrw) from Bacillus subtilis and BT3352 (4dwo) from Bacteroides thetaiotaomicron, with z scores of 16.6 and 16.5, respectively. Although Sav2152 has 22%, 25%, and 23% sequence identities with YigL, YwpJ, and BT3352, respectively, these contain four short HAD signature motifs known to be possessed by HAD-like phosphatases (Figure 3A). Moreover, the structure comparison of Sav2152 with these three proteins reveals that the tertiary structures of the core region are relatively well matched to each other with overall root-mean-square deviations (RMSDs) of 1.727, 1.814, and 2.195 Å, respectively (Figure 3B and Figure S2). However, a partial region matching α5 and α6, which belong to the cap domain of Sav2152, shows diversity among HAD-like phosphatases, as shown in Figure 3C. BT3352 has a structure where a Mg^2+^ ion is coordinated with enzymatic key residues. Interestingly, the structures of YigL and YwpJ were determined with the coordination of a Ca^2+^ ion instead of Mg^2+^. The key residues coordinating Ca^2+^ and Mg^2+^ in the structures of YigL and BT3352, respectively, were compared to the matching residues in the structure of Sav2152 to characterize the functional key residues (Figure 3D). A comparison of enzymatic key residues indicates that D10, D12, and D233 in Sav2152 structurally correspond to D8, D10, and D214, coordinating Ca^2+^ in the structure of YigL, and D9, D11, and D219, coordinating Mg^2+^ in the structure of BT3352, respectively. Although these key residues match relatively well, there may be a slight difference in the orientation of the side chain in Sav2152, possibly due to the absence of metal ions. The B-factor values (Figure 3E) indicate that D12 and D233 in Sav2152 exhibit local dynamics. 

The presence of C2-type cap modules is known to sterically restrict access to the catalytic cavity, allowing C2-type phosphatases to act on small molecules. In this reaction, the enzymatic area is isolated within the active site by cap closure and exhibits substrate specificity [20]. Enzymatic functional residues of HAD phosphatase are located in a cavity between the core domain and the cap domain. The structure of Sav2152 reveals that α5 and α6 in the cap domain may play an important role in substrate specificity because α5 and α6 can make an isolated space in the active site with the core domain. As shown in Figure 3F, the electro-potential map of Sav2152 demonstrates that negatively charged residues are clustered around the enzymatic region in the core domain, while the cleft’s deep inside region and the cap region close to enzymatic residues consist of positively charged residues. The electro-potential maps of BT3352 and YwpJ show similar but not identical charge distributions around the activity site. This difference can contribute to substrate specificity. 

### 2.4. Enzyme Activity of Sav2152

**Figure 4:**
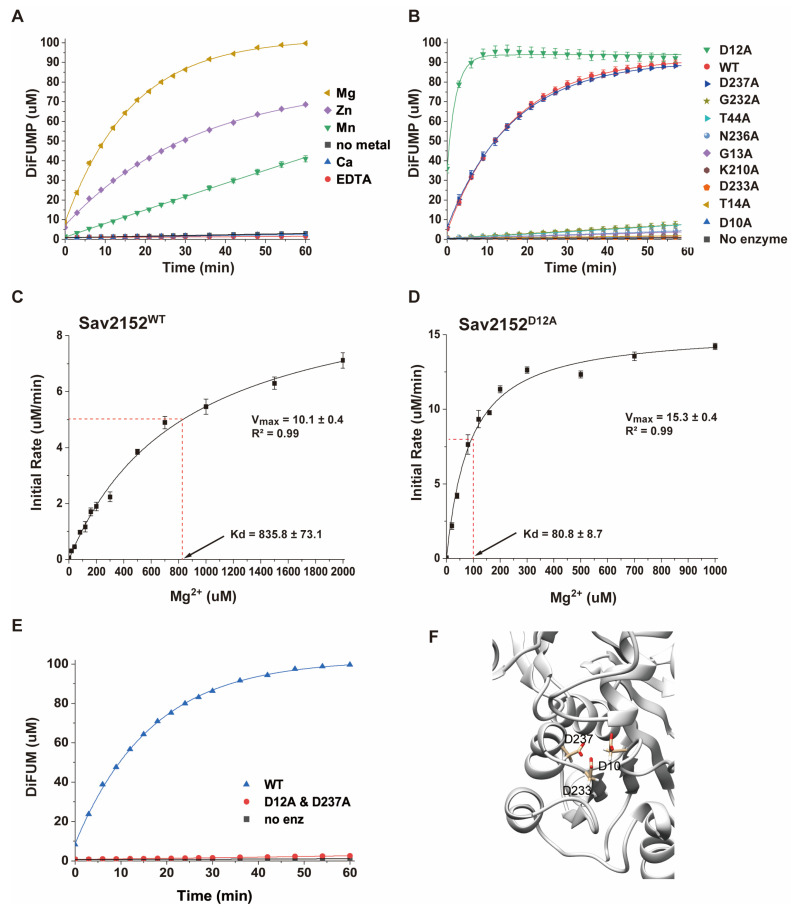
Phosphatase activities of Sav2152. (**A**) Metal ion-dependent phosphatase activity using Mg^2+^, Zn^2+^, Mn^2+^, Ca^2+^, and EDTA. (**B**) Phosphatase activities of mutants Sav2152^D10A^, Sav2152^D12A^, Sav2152^G13A^, Sav2152^T14A^, Sav2152^T44A^, Sav2152^K210A^, Sav2152^G232A^, Sav2152^D233A^, Sav2152^N236A^, and Sav2152^D237A^. The phosphatase activity of Sav2152^D12A^ was increased, and the phosphatase activity of Sav2152^D237A^ is similar to that of wild-type Sav2152. The order of mutants in the figure corresponds to the top-to-bottom arrangement in the graphs. (**C**,**D**) The initial rates at different Mg^2+^ ion concentrations for enzymatic reactions by Sav2152^WT^ (**C**) and Sav2152^D12A^ (**D**). (**E**) The phosphatase activity of the double-mutant Sav2152^D12A^ and ^D237A^. (**F**) The structural coordination of D10, D233, and D237 on the modeling structure. Residues D10, D233, and D237 potentially coordinate with the metal ion. All experiments were conducted in the presence of 10 μM of the protein and 1 mM Mg^2+^ ions, and all measurements were performed in triplicate.

Since the structure of Sav2152 was determined without a metal ion, the enzymatic activity of this protein was investigated using a simple phosphatase assay kit with DiFMUP as a phosphate template. HAD phosphatases are well known to be Mg-dependent enzymes, so we studied the efficiency of the enzymatic activity of Sav2152 with and without a divalent ion such as Zn^2+^, Mn^2+^, Ca^2+^, or Mg^2+^. As shown in Figure 4A, a Mg^2+^ ion is indeed critical for its enzymatic function, while Zn^2+^ and Mn^2+^ ions, although less efficient than Mg^2+^, also facilitate enzyme activity. However, Ca^2+^ ions could not activate Sav2152. To understand the detailed mechanism of phosphate cleavage, the enzymatic activity of Sav2152 containing single-residue mutations was examined. In this experiment, 10 highly conserved amino acids in the top homologs were replaced with alanine: D10A, D12A, G13A, T14A, T44A, K210A, G232A, D233A, N236A, and D237A. The resulting Sav2152 mutants with single-site mutations are referred to as a Sav2152 mutation site. Residues D10, D12, G13, and T14 belong to motif I; T44 to motif II; K210 to motif III; and G232, D233, N236, and D237 to motif IV. D10 and D12 in motif I are usually directly involved in catalytic reactions. T44 in motif II and K210 in motif III were predicted to help stabilize the substrate for the catalytic reaction. D233 and D237 in motif IV are typically involved in coordination with Mg^2+^ ions. Interestingly, the alanine mutation screening yielded unexpected results. As expected, the enzymatic activity of most mutations was significantly abolished (Figure 4B). However, the phosphatase activity of Sav2152^D12A^ and Sav2152^D237A^ was not abolished, and in the case of Sav2152^D12A^, it was even increased. To determine the binding affinity between Sav2152 and Mg^2+^ ions and the effect of D12A mutation on this binding, the initial rates of the proteins on ligands at various concentrations of the Mg^2+^ ions were measured (Figure 4C,D and Figure S3). The results are fitted to a single-site binding equation. As shown in Figure 4C,D, the predicted maximum velocities (V_max_) of Sav2152^WT^ and Sav2152^D12A^ are 10.1 ± 0.4 and 15.3 ± 0.4, respectively. The values of the dissociation constant (K_d_) of Sav2152^WT^ and Sav2152^D12A^ were calculated as 835.8 ± 73.1 and 80.8 ± 8.7 μM, respectively, which indicates that Sav2152^WT^ is considered to be a phosphatase with weak activity. To identify a residue that can replace D12 and binds to Mg^2+^ ions, the phosphatase activity of Sav2152^D12A and D237A^ was measured (Figure 4E). The result showed that the enzymatic activity of Sav2152^D12A and D237A^ almost disappeared. As shown in Figure 4F, D10, D233, and D237 were found to be sufficiently close to coordinate Mg^2+^ ions in the Alphafold model of Sav2152.

### 2.5. Thermal Stability of Sav2152

**Figure 5:**
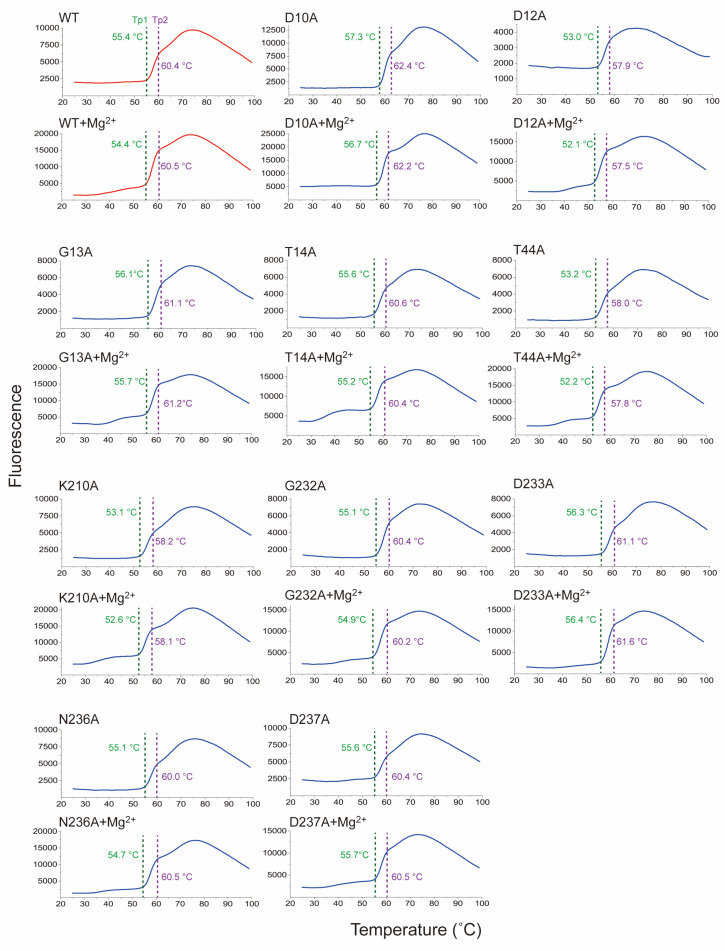
The structural transition of Sav2152 and its mutants. Tp1 and Tp2 transitions are indicated by a green and purple dotted line, respectively. The sample solutions for the thermal shift assay contained 2.9 μM of each protein and 30 μM of Mg^2+^ ions. The measurements were performed in triplicate.

To understand the structural characterization of Sav2152, we investigated changes in thermal stability in response to mutations and the presence of metal ions, through melting temperature (Tm). Tm represents the temperature at which the protein undergoes folding or unfolding transitions or different conformational states. To analyze the results of the thermal shift assay, we initially calculated the Boltzmann Tm values from the inflection point of the fluorescence plot. However, this approach is incorrect because the curve presents multiple transitions. Therefore, we manually identified the point where each transition is considered to occur. Sav2152^WT^ and other mutated variants exhibited two distinct transitions points, subsequently referred to as Tp1 and Tp2, respectively (Figure 5, Figure S4, and Table 1). As a result, each mutation is suggested to contribute differently to the Tp1 and Tp2 of the protein. For example, the Tp1 and Tp2 values of Sav2152^D12A^ significantly decreased when compared to Sav2152^WT^. On the other hand, Sav2152^D10A^ and Sav2152^D233A^ showed an increase in Tp1. Interestingly, Sav2152^D12A^, which exhibited enhanced enzymatic activity, showed the most significant decrease in Tp1 and Tp2 values, when compared to those of Sav2152^WT^. Additionally, in experiments investigating changes in Tp1 and Tp2 due to metal binding, unexpected results were observed. The coordination of Mg^2+^ ions into the catalytic cavity resulted in a small transient shoulder just before Tp1 in the thermal shift plot except Sav2152^D10A^. Meanwhile, Tp1 and Tp2 appeared to be unaffected by the presence of metal ions.

## 3. Discussion

In this study, the crystal structure of Sav2152 without a metal ion was determined, but a partial C-terminal region, including two conserved residues in motif IV, was missing. It is important to note that the metal-free structure is not considered a physiological form within the cell, as Mg^2+^ ions are known to exist in concentrations exceeding a single-millimolar range within the cell [22]. Despite extensive efforts, we could not obtain crystals of the holo form of Sav2152 containing a metal co-factor. These limit our understanding of the protein’s function based on its structure. To compensate for this limitation, a comparison was made with structures that have similar features. The focus was on the enzymatic key residues that were expected to bind to a metal ion and directly participate in the cleavage reaction.

**Figure 6:**
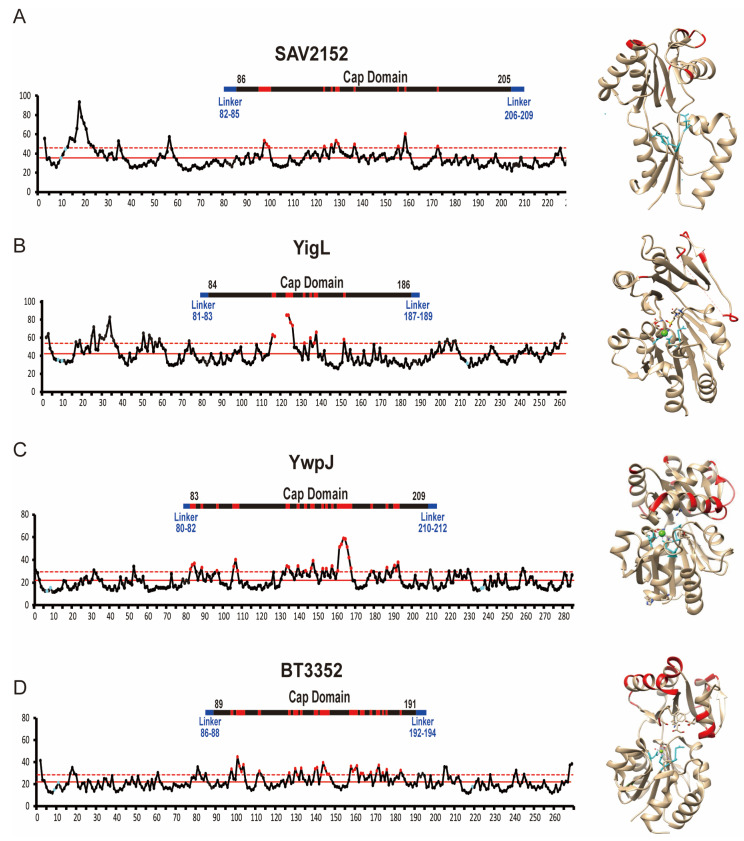
A B-factor analysis of Sav2152, YigL, YwpJ, and BT3352. A B-factor plot and the structure of Sav2152 (**A**), YigL (**B**), YwpJ (**C**), and BT3352 (**D**). The residues predicted to play a crucial role in metal coordination are highlighted in blue on the B-factor plot and within the protein structure. The residues that exceed the upper standard deviation from the mean are highlighted in red both on the B-factor plot and within the protein structures.

Based on the previous reports, the first highly conserved residue, D10, in Sav2152 serves as a nucleophile [23], while the second residue, D12, located two residues C-terminal to the first nucleophile, acts as a general acid/base catalyst [20]. These residues, along with D233, were predicted to be involved in the coordination of Mg^2+^ ions. Indeed, the structures of other proteins such as BT3352 and YigL revealed that corresponding residues coordinate with Mg^2+^ and Ca^2+^ ions, respectively. Furthermore, these structures showed that residues around the metal ion are stabilized with low B-factor values (Figure 6). However, in the structure of Sav2152 without a metal ion, the side chains of D12 and D233 exhibited high B-factor values, indicating local dynamic movement. D12 and D233 in Sav2152 may undergo movement to bind with a metal ion and become stabilized. Therefore, metal ions appear to be involved not only in enzymatic function but also in stabilizing enzymatic regions. Given that the wild type of Sav2152 has a phosphatase activity dependent on the metal ion, it can fold adequately with a metal ion into the active form. On the other hand, it was found that Mg^2+^ ions have a contradictory effect on proteins. The Mg^2+^ ion has been known to stabilize catalytic residues. In contrast, our temperature shift assays results of Mg^2+^-containing Sav2152 wild-type and other mutants, which exhibited structural transition occurring before Tp1, indicate that Sav2152 binding with the Mg^2+^ ion can also result in structural destabilization. Therefore, it is possible that crystals of the metal-bound form of Sav2152 could not be obtained due to structural destabilization caused by metal binding. However, considering the minimal change in fluorescence, this effect is likely attributed to changes in the local structure. C2-type phosphatases have ‘substrate specificity domains’ in the caps [15,24]. Thus, the substrate specificity domains of Sav2152 might be α5 and α6. The residues in these domains typically interact with the substrate-leaving group and activate the substrate for nucleophilic attack [20]. Metal ions are likely to influence local movements, promoting easier binding with specific substrates. However, no dramatic conformational changes, such as an open–close form exchange, were observed in the structural comparison of homologs (Figure 3B). Nevertheless, interestingly, a B-factor analysis of Sav2152 and its homologs reveals that YigL, YwpJ, BT3352, and Sav2152 have high B-factor values that exceed the standard deviation, with 16 out of 103, 30 out of 127, 29 out of 103, and 11 out of 120 residues within the Cap domain, respectively (Figure 6). In other words, it appears that the atomic mobility of the Cap domain in the metal-binding structure seems to be higher than that of Sav2152. Due to the limitations of the Sav2152 structure, which lacks a visible C-terminus and does not bind to metal, it is difficult to definitively state that the instability of the Cap domain arises from metal binding. However, the potential for such a relationship can be suggested, and this aspect warrants further investigation in the future.

Residue D12 is typically considered a key residue for enzymatic activity in HAD-like phosphatase. Nonetheless, it is worth noting that an enzymatic activity experiment of Sav2152^D12A^ demonstrated 10 times higher affinity with Mg^2+^ and more efficient phosphate cleavage compared to wild-type Sav2152. These surprising results appear to be closely related to the binding between aspartic acids and metal ions. Unfortunately, we are unable to determine the number of binding sites between Sav2152 and Mg^2+^ ions. However, considering the structural homologies of Sav2152, which typically coordinate a single metal ion, and the mutations in conserved residues except D12 and D233, which severely impair enzymatic activity, it is likely that Sav2152 indeed coordinates with one metal, which is consistent with the fact that points that resulted from the initial rate of Sav2152^WT^ at a various concentration of Mg^2+^ ions were well fitted to a single binding equation with an R2 value of 0.99 (Figure 4C). Consequently, the increase in the activity of Sav2152^D12A^ can be explained by the functional substitution of D12, which acts as a base catalyst, with another residue. Hence, two negatively charged residues in the vicinity of D12, namely D235 and D237, were considered potential replacements. However, a double-alanine mutant involving D12 and D235 was not able to abolish the phosphatase activity (data are not shown), indicating that D235 cannot replace D12 effectively. The phosphatase assay of Sav2152^D12A and D237A^ confirmed that the activity significantly decreased (Figure 4E), supporting the idea that D237 could potentially replace D12. Despite the crystal structure lacking the C-terminal region, computational modeling predicted that D237 could coordinate with the metal ion in conjugation with D10 and D233 (Figure 4F). In summary, D10, D12, and D233 are involved in the coordination of the Mg^2+^ ion in Sav2152^WT^; however, the binding affinity and enzymatic efficiency are low; on the other hand, D10, D233, and D237 can coordinate with the Mg^2+^ ion in Sav2152^D12A^, leading to an increase in both binding affinity and enzymatic efficiency. The enzymatic reactions of Sav2152^WT^ and Sav2152^D237A^ look similar, as shown in Figure 4B, which reveals that D237 is not significantly involved in the enzyme-catalyzed reaction. However, when D12 is replaced with Ala, D237 can finally bind to the Mg^2+^ ion and engage in a more efficient enzymatic reaction. In order to understand the exact mechanism by which the enzymatic activity of Sav2152^D12A^ increases, the structures of Sav2152^WT^ and Sav2152^D12A^ including a metal ion, are needed, but unfortunately these structures could not be obtained. Regardless, based on the observed decrease in Tp1 and Tp2 for Sav2152^D12A^, it is evident that D12 plays a pivotal role in stabilizing Sav2152. The mutation of D12 to alanine destabilizes the structure, which may lead to local structural changes around the activity cavity, intriguingly, allowing the Mg^2+^ ion to access D237 and making it easier for substrates to enter the active site. This explains why the phosphatase activities of Sav2152^D12A^ and Sav2152^D237A^ were not decreased, unlike other conserved residues. Considering the work conducted by Kuznetsova et al., which demonstrated that HAD phosphatase mutations of *Saccharomyces cerevisiae* corresponding to the D12 of Sav2152 completely lost their enzymatic activity [25], it was deduced that this replacement is not a common occurrence in the case of HAD phosphatase. These findings suggest that Sav2152 possesses a unique system for maintaining activity in diverse environments.

As mentioned above, D12 is involved in stabilizing Sav2152. However, the decrease in both the Tp1 and Tp2 of Sav2152^D12A^ does not seems to be associated with the destabilizing mechanism by Mg^2+^ ions, as the first thermal transition was still observed in the temperature shift assay. In contrast, the structural transition before at Tp1 was not observed in the result of Mg^2+^-containing Sav2152^D10A^, unlike other metal-containing Sav2152 proteins. Additionally, given that the Tp1 and Tp2 values of Sav2152^D10A^ remain unchanged regardless of metal presence and considering the loss of the phosphatase activity of it, it seems that the D10A mutant is unable to bind to Mg ions. Consequently, this suggests that D10 is essential for binding to Mg^2+^ ions.

Taken together, it is believed that metal ions likely trigger an intermediate state of Sav2152, facilitating efficient substrate binding. Subsequently, the substrate stabilizes the intermediate state. Sav2152 is a weak metal-dependent enzyme but possesses a remarkable latent enzymatic system. However, this study had a limitation in that the metal-free structure of Sav2152 is non-physiological within the cell where Mg^2+^ ions are known to exist in concentrations exceeding a single-millimolar range. Thus, the metal-bound structure is required for prospective studies in the future.

## 4. Materials and Methods

### 4.1. Protein Preparation

The cDNA of Sav2152 from *S. aureus* strain Mu50 was amplified using polymerase chain reaction (PCR). The amplified cDNA was then inserted into the pET-28a vector. The vectors were transformed into E. coli BL21-Codon Plus (DE3)-RIL. A single colony was inoculated into 0.5 L of Luria–Bertani (LB) medium supplemented with antibiotics and grown at 37 °C with vigorous shaking for 6 h. When OD600 reached 0.65, 1 mM IPTG was added, followed by incubation at 24 °C for 24 h. The cells were harvested and resuspended in 20 mL of lysis buffer (25 mM Tris–HCl pH 7.0, 300 mM NaCl, 13 mM 2-mercaptoethanol, and 1 mM cocktail inhibitor) per gram of wet cells. The suspension was sonicated for 10 min with a 50% duty cycle using an Ultrasonic homogenizer KUH-650 (KBT, Kyungkido, Republic of Korea). Inclusion bodies were pelleted by centrifugation at 14,800 g for 40 min and discarded. The supernatants were applied to Ni-NTA resin (1.2 mL/g of cells). The Ni-NTA resin was washed with a buffer containing 40 mM imidazole and then eluted with a buffer containing 250 mM imidazole. The eluted solution was desalted and applied to a 16/600 SuperdexTM (Cytiva, Marlborough, MA, USA.) gel filtration column equilibrated with a buffer (25 mM Tris HCl, pH 7.0, 100 mM NaCl). The protein solution was eluted with buffer B and concentrated to 20 mg/mL using a 30 kDa Amicon (Merck Millipore Inc., Burlington, MA, USA).

### 4.2. Mutagenesis in Sav2152 

The pET-28a vector in method 2.1, which contains the Sav2152 sequence, was used as a template DNA for mutagenesis. Amino acids that play a key role in phosphatase activity (D10A, D12A, G13A, T14A, T44A, K210A, G232A, D233A, N235A, and D236A) were mutated to alanine. The residues were mutated using the QuickChange site-directed kit (Agilent, Santa Clara, CA, USA).

### 4.3. Multiple-Angle Light Scattering

The oligomeric state of Sav2152 was analyzed by multiple-angle light scattering using a Dawn Heleos Ⅱ (18 angles), Optilab T-Rex (RI) [Wyatt Technology, Santa Barbara, CA, USA] instrument. Sav2152 at a concentration of 1.0 mg/mL in a buffer (25 mM Tris-HCl, pH 7.0, 100 mM NaCl) was centrifuged at 14,800 g for 20 min, and the supernatant was used for data collection. The data were taken at 298K. The regularization histogram was analyzed using ASTRA 6.1.7.17 software.

### 4.4. Crystallization and Data Collection

Crystallization was performed by the vapor diffusion method using 1 μL of a 20 mg/mL protein solution with an equal volume of reservoir solution at 20 °C. The initial screening of crystallization conditions was performed using the wizard crystallization screen kit (Ⅰ, Ⅱ, Ⅲ, and Ⅳ) with mosquito Xtal3 (SPT Labtech, Boston, MA, USA). Crystals were obtained using a reservoir solution consisting of 100 mM CAPS, pH 10.5, 13% PEG 8000, and 400 mM NaCl. The cryoprotectant solution for cooling the crystal consisted of 100 mM CAPS pH 10.5, 13% PEG 8000, 400 mM NaCl, and 20% (*v*/*v*) glycerol. The crystal belonged to space group P21 2 21 with unit cell dimensions of a = 53.61, b = 63.06, and c = 92.73 Å and diffracted to approximately 1.92 Å. Diffraction data sets were collected at Pohang Accelerator Laboratory (PAL) BL-5C beamline, Pohang, Korea. All data sets were processed with X-ray Detector Software (XDS, Version Feb. 5, 2021) [26]. 

### 4.5. Structure Determination

The alphafold2 model obtained from the Colabfold website (https://colab.research.google.com/github/sokrypton/ColabFold, accessed on 5 November 2021) was used for MR. The N-terminus of the predicted structure (residues 1–83) was used as a template for MR. MR was performed using the program Phaser included in the PHENIX suite [27]. The Sav2152 structure was manually rebuilt in Coot and iteratively refined in PHENIX. After rebuilding, water molecules were added using the Coot program [28]. All residues, except one of Sav2152, were in the preferred region of the Ramachandran plot.

### 4.6. Phosphatase Activity Measurement

The phosphatase assay was conducted with the EnzCheck Phosphatase Assay Kit (Molecular Probes, Eugene, OR, USA). The 100 μL reaction mixture in the 96-well black plate for the measurement of phosphatase activity consisted of a 100 μM 6,8-difluoro-4-methylumbelliferyl phosphate (DiFMUP) substrate, 1 mM metal such as Mg^2+^, Zn^2+^, Mn^2+^, or Ca^2+^, 100 mM NaCl, 25 mM Tris-HCl pH 7.0, and a 10 μM protein sample. The reaction was started by adding the protein, and the results were measured with the Filter Max F3 (Molecular Devices, San Jose, CA, USA) as soon as possible after adding the protein to the reaction mixture. The interval of measurement was 3 min. The amount of the product, 6,8-difluoro-4-methylumbelliferyl (DiFMU), in the solution was determined photometrically at 460 nm. The graph for the phosphatase assay was modified using Origin 2022 (OriginLab, Northampton, MA, USA). The data were fitted to the quadratic equation to calculate a Kd value between Mg^2+^ and Sav2152, as follows:

$$V=V_{max}\times \frac{\left[P_{total}\right]+\left[A_{total}\right]+Kd-\sqrt{{\left(\left[P_{total}\right]+\left[A_{total}\right]+Kd\right)}^{2}-4\times \left[P_{total}\right]\times [A_{total}]\times Kd}}{2[P_{total}]}$$

where V represents the initial reaction velocity, V_max_ represents the maximum initial reaction velocity, P_total_ represents the total amount of protein, A_total_ represents the total amount of Mg^2+^ ion, and Kd represents a dissociation constant. 

### 4.7. Melting Temperature Scanning

The reaction mixture for the melting temperature scanning consisted of 20 μL and included the following components: 5 μL of Protein Thermal ShiftTM Buffer, 2.5 μL of Diluted Protein Thermal ShiftTM Dye (8X), and 12.5 μL of a buffer solution (25 mM Tris-HCl, pH 7.0, 100 mM NaCl) containing 2 μg of Sav2152 and 30 μM of metal. The reaction solutions (10 μL) were filled in the wells of a 384-well plate. The plate was measured using QuantStudio 5 (Applied Biosystems, Waltham, MA, USA) with the temperature rising from 25 to 99 °C at an increase of 0.5 °C/s. The Protein Thermal Shift^TM^ Dye bound to the denatured part of Sav2152 was caused by heat, which was determined by fluorescence intensity. The data analysis was performed using the Protein Thermal Shift^TM^ Software ver.1.4

## Figures and Tables

**Table 1:** Data collection and refinement statistics.

**Data Collection**	
wavelength (Å)	1.0000
resolution (Å)	27.76–1.92 (1.99–1.92)
space group	*P* 2_1_ 2 2_1_
unit cell dimensions	
*a*, *b*, *c* (Å)	53.61, 63.06, 92.73
α, β, γ (°)	90, 90, 90
No. of total reflections	177,406 (17,205)
No. of unique reflections	24,627 (2409)
completeness (%)	99.83 (99.88)
Mean *I*/sigma (*I*)	14.24 (4.99)
*R*_merge_ ^a^	0.0823 (0.4843)
*R*_meas_	0.08952 (0.5221)
*R*_pim_ ^b^	0.03321 (0.1922)
CC1/2	0.996 (0.958)
**Refinement**	
Reflections used in refinement	24,638 (2408)
Reflections used for *R_free_*	1231 (121)
*R_work_*/*R_free_* (%) ^c^	0.1957 (0.2243)/0.2318 (0.2994)
No. of Protein residues / *B* factors (Å^2^)	231/35.90
No. of Water / *B* factors (Å^2^)	147/41.85
Wilson B-factor	29.10
rmsd (bonds)	0.013
rmsd (angles)	1.33
Ramachandran favored (%)	97.82
Ramachandran allowed (%)	1.75
Ramachandran outliers (%)	0.44
Rotamer outliers (%)	0.99
Clashscore	4.38

Values in parentheses are for the highest-resolution shell; a *R_merge_* = ∑hkl ∑i |Ii(hkl) − <I(hkl)> | / ∑hkl ∑iIi(hkl). b *R_p.i.m_*. is defined as ∑hkl{1/[N(hkl) – 1]}1/2 ∑i |Ii(hkl) – I(hkl)| / ∑hkl ∑iIi(hkl). c *R* = Σhkl ||Fobs| − k |Fcalc||/ Σhkl |Fobs|, where Rfree was calculated by the same way as Rwork.

## Data Availability

The crystal structure has been deposited into the Protein Data Bank with accession codes 7XHZ.

## Supplementary Materials

The following supporting information can be downloaded at: https://www.mdpi.com/article/10.3390/ijms25095021/s1.
