# Supplementary figures and images for: Nontraditional Roles of Magnesium Ions in Modulating Sav2152: Insight from a Haloacid Dehalogenase-like Superfamily Phosphatase from Staphylococcus aureus
Source: Int J Mol Sci. 2024 May 4;25(9):5021. doi: 10.3390/ijms25095021 (PMC11084212; doi:10.3390/ijms25095021)

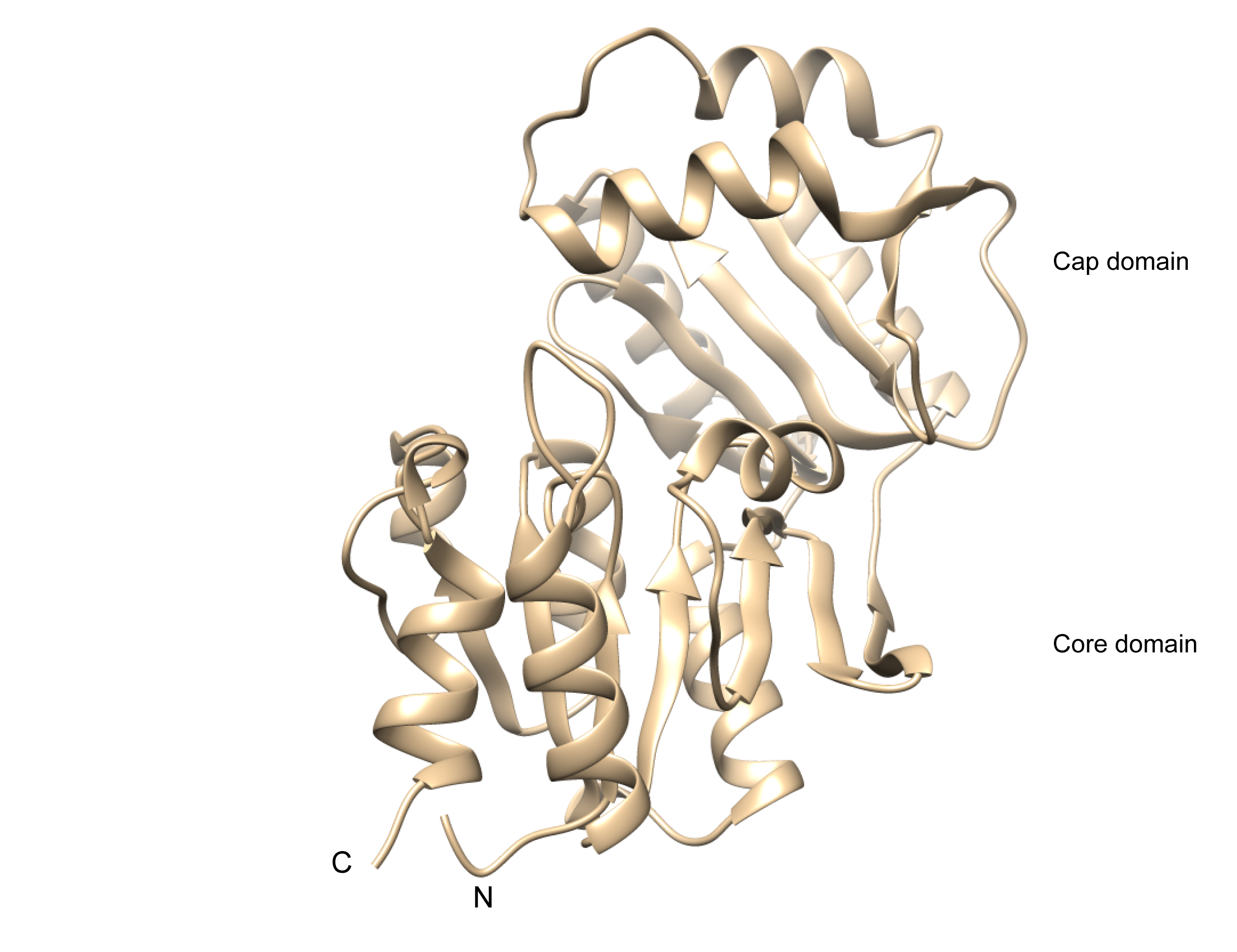

Supplement: Supplementary file 1 [file ijms-25-05021-s001.zip › FigureS1.tif]

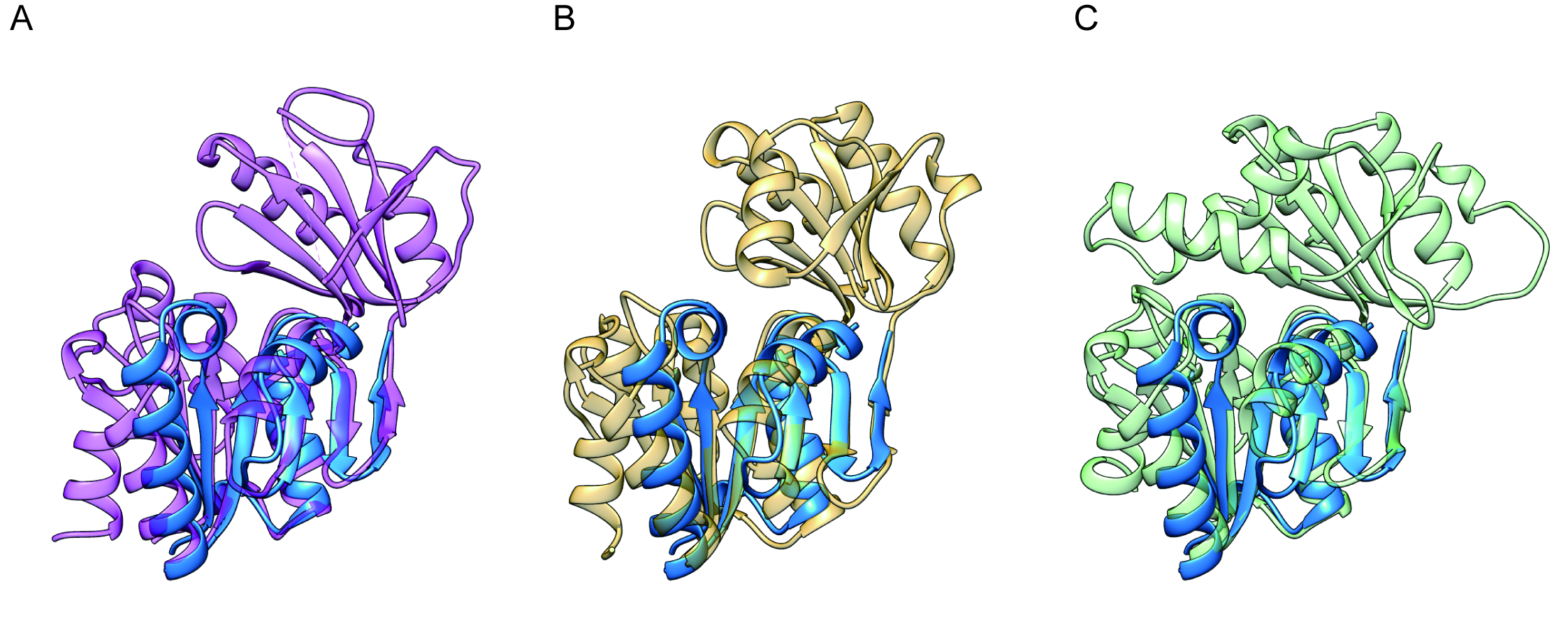

Supplement: Supplementary file 1 [file ijms-25-05021-s001.zip › FigureS2.tif]

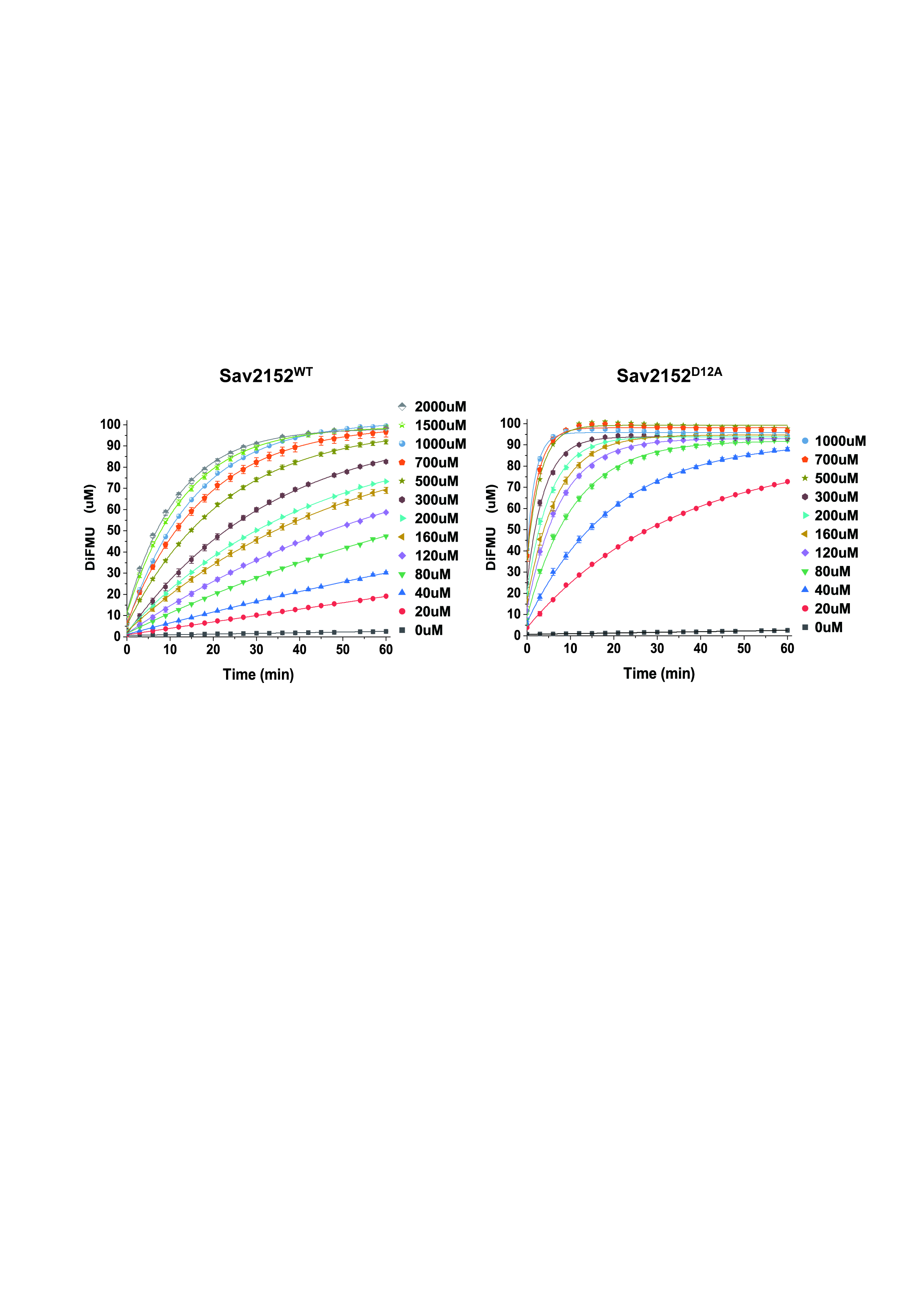

Supplement: Supplementary file 1 [file ijms-25-05021-s001.zip › FigureS3.tif]

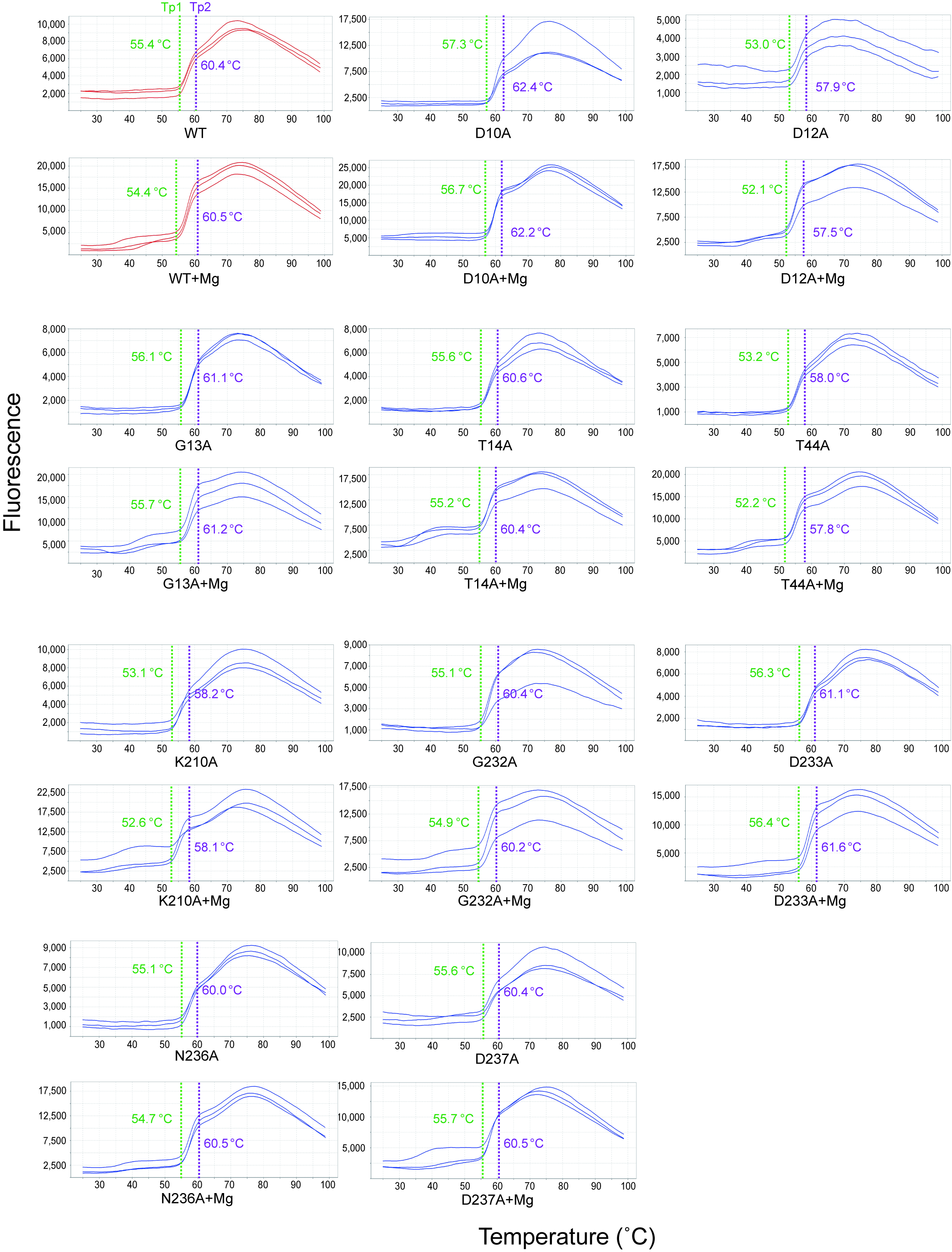

Supplement: Supplementary file 1 [file ijms-25-05021-s001.zip › FigureS4.tif]
